# A Rare Case of Bladder Cancer With Squamous Differentiation Causing Hypercalcemia and Leukocytosis

**DOI:** 10.7759/cureus.80111

**Published:** 2025-03-05

**Authors:** Allison T Yip, Shalini Bhat

**Affiliations:** 1 Endocrinology, Diabetes, and Metabolism, University of California Los Angeles David Geffen School of Medicine, Los Angeles, USA; 2 Division of Endocrinology, Diabetes, and Hypertension, Veterans Affairs Greater Los Angeles Healthcare System, Los Angeles, USA

**Keywords:** bladder cancer, humoral hypercalcemia of malignancy, hypercalcemia leukocytosis syndrome, hypercalcemia of malignancy, paraneoplastic leukocytosis, parathyroid hormone-related protein (pthrp), squamous cell carcinoma

## Abstract

The most common cause of humoral hypercalcemia of malignancy is excessive secretion of parathyroid hormone-related peptide (PTHrP), which can include solid tumors, most commonly squamous cell carcinoma (SCC). It is unusual to see hypercalcemia associated with bladder cancer and local tumor-related secretion of PTHrP from SCC of the bladder. In addition to hypercalcemia, leukocytosis can be a concomitant neoplastic syndrome resulting from cytokine production of granulocyte colony-stimulating factor (G-CSF) from poorly differentiated cells. We discuss a case of a 62-year-old male who came to medical attention after a workup for weight loss, fatigue, and urinary retention revealed a new bladder mass, sepsis from a urinary source, and hypercalcemia. His hypercalcemia was initially managed with calcitonin, bisphosphonates, and intravenous (IV) fluids until surgery, after which the serum calcium briefly normalized and his leukocytosis reached its nadir. Pathology identified poorly differentiated SCC. However, due to the locally invasive nature of the tumor, he had an incomplete resection and experienced rebound hypercalcemia and worsening leukocytosis.

This report highlights a rare case of poorly differentiated SCC of the bladder associated with the paraneoplastic syndrome of hypercalcemia and leukocytosis, both of which corresponded to poor prognosis. Managing PTHrP-mediated hypercalcemia requires both the correction of the immediate metabolic disturbance and treatment of the underlying malignancy; lastly, the persistent leukocytosis in this case was not associated with sepsis or infection and likely correlated with the incomplete eradication of the malignancy.

## Introduction

Hypercalcemia of malignancy can occur in up to 20-30% of all cancer patients [1]. Various mechanisms can cause hypercalcemia in malignancy, including lytic bone destruction through metastasis, tumor production of parathyroid hormone-related peptide (PTHrP), and tumor production of 1,25-dihydroxyvitamin D (calcitriol). Hypercalcemia of malignancy is associated with hematologic malignancies like multiple myeloma, or solid tumors, most commonly breast, renal, or squamous cell carcinoma (SCC) of any origin [2]. SCC of the bladder is a rare malignancy, constituting less than 5% of all bladder cancers in the United States [3]. SCC of the bladder has significant geographic variability in incidence and is common in regions with endemic schistosomiasis. In non-endemic areas, SCC of the bladder is often associated with chronic irritation or inflammation, including recurrent urinary tract infections, prolonged catheterization, or bladder stones. Unlike urothelial (otherwise known as transitional cell carcinoma (TCC)], which dominates bladder cancer diagnoses, SCC is the second most common histological subtype; it tends to present at a more advanced stage and is independently associated with a worse prognosis [3,4].

In general, it is infrequent for any histological subtype of localized bladder cancer to be associated with paraneoplastic syndromes [5]. Occasionally, hypercalcemia alone has been reported, and, in these cases, it typically resolves after treatment of the bladder cancer [6-8]. Bladder cancers with paraneoplastic granulocyte colony-stimulating factor (G-CSF) production have been associated with marked leukocytosis, which is linked to high-grade histology and presents as a more advanced disease [9,10]. In a case report by Khawaja et al., a literature review of 35 patients with leukocytosis associated with bladder cancer revealed that TCC was the most frequent histology with nine patients having associated hypercalcemia, six of which had documented elevated PTHrP [10].

Although uncommon, this combination of paraneoplastic leukocytosis and hypercalcemia in bladder cancer was described as early as 1973 by Block et al. [11], and then again in 1986 by Bennett et al. [12] in their case series, in which two out of four patients had both hypercalcemia and leukocytosis. The current review of the literature revealed five cases of this hypercalcemia-leukocytosis syndrome associated with SCC of the bladder, two of which were pure SCC histopathology [13,14] while the remaining three cases were of mixed TCC+SCC pathology [15-17]. In this report, we describe the third case, involving a patient who presented with hypercalcemia and leukocytosis due to a poorly differentiated SCC of the bladder.

## Case presentation

A 62-year-old male with a past medical history of non-ST-elevation myocardial infarction (NSTEMI) and hyperlipidemia originally presented to the urology clinic with a one-month report of urinary retention and gross hematuria requiring workup. Before the presentation, the patient reported being very active, usually biking 10-20 miles per day. He had a one-pack-year smoking history and quit 39 years ago. He had recently lost 20 pounds over the past two months. He underwent an outpatient CT urogram, which revealed a large 7 cm bladder diverticulum with the entirety filled with tumor, followed by cystoscopy with cytopathology of the bladder wash, raising concerns for malignancy.

One week later, he was admitted to an outside hospital for syncope and fatigue and was found to have sepsis from a urinary source, a large peri-vesicular fluid collection, acute kidney injury, acute deep vein thrombosis (DVT), and hypercalcemia of malignancy. His left common femoral vein DVT was managed with inferior vena cava (IVC) filter placement due to hematuria while on a heparin drip. Interventional radiology placed an anterior abdominal drain into the peri-vesicular fluid collection and cultures grew Veillonella parvula; and he was treated with intravenous (IV) antibiotics. At this outside hospital, labs revealed calcium of 15.0 mg/dL (8.5-10.0 mg/dL), albumin of 2.6 g/dL (3.5-4.8 g/dL), and creatinine of 2.06 mg/dL (0.6-1.3 mg/dL). Before hospitalization, he had normal renal function. He received pamidronate and four doses of calcitonin, and the calcium improved to 8.7 mg/dL and albumin to 1.5 g/dL, which was still in the range of mild hypercalcemia when corrected for his low albumin.

**Figure 1:**
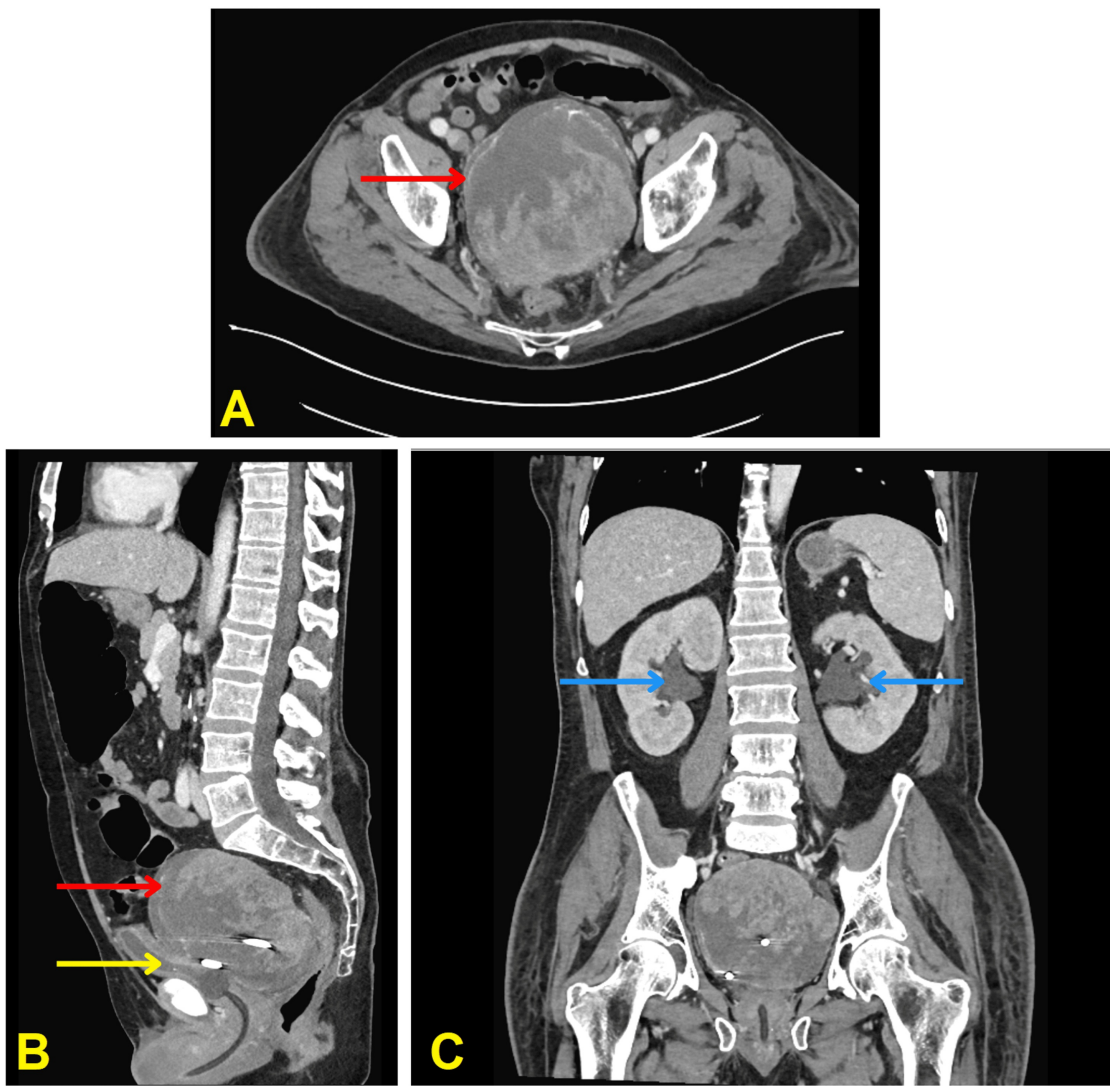
CT scan of abdomen and pelvis with contrast (A) axial view, (B) sagittal view, and (C) coronal view. The urinary bladder is collapsed (yellow arrow in panel B) with a Foley catheter in place. Complex fluid collection corresponding with internal density/mass lesion, with an anterior approach suprapubic drainage catheter. The catheter terminates in the complex fluid collection which measures 12.7 cm AP by 11.4 cm transverse by 10.4 cm craniocaudal (red arrow in panels A, B). Bilateral pelviectasis and hydroureter (blue arrows in panel C) CT: computed tomography

He was transferred to the West Los Angeles Veterans Affairs Medical Center two weeks later for definitive treatment for his bladder mass (Figure 1), which had an interval increase in size. He was also noted to have hypercalcemia on admission, whereupon he was given zoledronic acid 4 mg IV along with four doses of calcitonin every 12 hours at 4 IU/kg.

Table 1 summarizes the patient's laboratory values on admission.

**Table 1:** Labs on admission PTH: parathyroid hormone; PTHrP: parathyroid hormone-related peptide; WBC: white blood cells

Variable	Patient value	Reference range
Calcium	10.6	8.4–10.2 mg/dL
Albumin	2.3	2.5–4.9 g/dL
PTH	11.4	14–72 pg/mL
Phosphorus	2.1	2.5–4.9 mg/dL
Creatinine	1.19	0.52–1.28 mg/dL
PTHrP	199	11–20 pg/mL
25-hydroxy vitamin D	26.2	30–100 ng/mL
1,25-dihydroxyvitamin D	36	18–72 pg/mL
WBC	28.53	4.50–11.00 K/uL
Neutrophil count	25120	1100–7700/uL
% Neutrophils	88.1	41.0–85.0%

Additional imaging for staging including CT chest, abdomen, and pelvis did not show any evidence of metastatic disease. There was a questionable low-attenuation focus within the T12 vertebral body, but an MRI cervical, thoracic, and lumbar spine study ruled out any focal, aggressive appearing, marrow-replacing osseous lesion, and the original lesion was favored to represent a hemangioma. 

The calcium level improved but never normalized despite IV fluids until the patient underwent surgical intervention. Urology recommended against any further drainage to prevent the risk of seeding malignant cells outside of the bladder. Urology believed that although the tumor appeared low grade on cystoscopy, due to the large tumor burden, transurethral resection of bladder tumor (TURBT) could cause risk of perforation, would unlikely to be curative, and require greater than five hours of operation time. Instead, due to the unique location and localization of the tumor, urology informed the patient and his family that it could be amenable to partial cystectomy or diverticulectomy with resection of the tumor-initiating cells along with the tumor with potential partial resection of his native bladder or conversion to open or a radical cystectomy.

The patient underwent a robotic-assisted diverticulectomy and partial cystectomy due to gross invasion into the rectum resulting in incomplete resection. His postoperative course was complicated by postop hemorrhage requiring massive transfusion protocol and emergent exploratory laparoscopy to achieve hemostasis at the surgical bed along with clot evacuation. The pathology revealed poorly differentiated squamous cell carcinoma, with lymphovascular invasion and positive surgical margins; it was deemed pathologic stage: pT3aNxMx.

**Figure 2:**
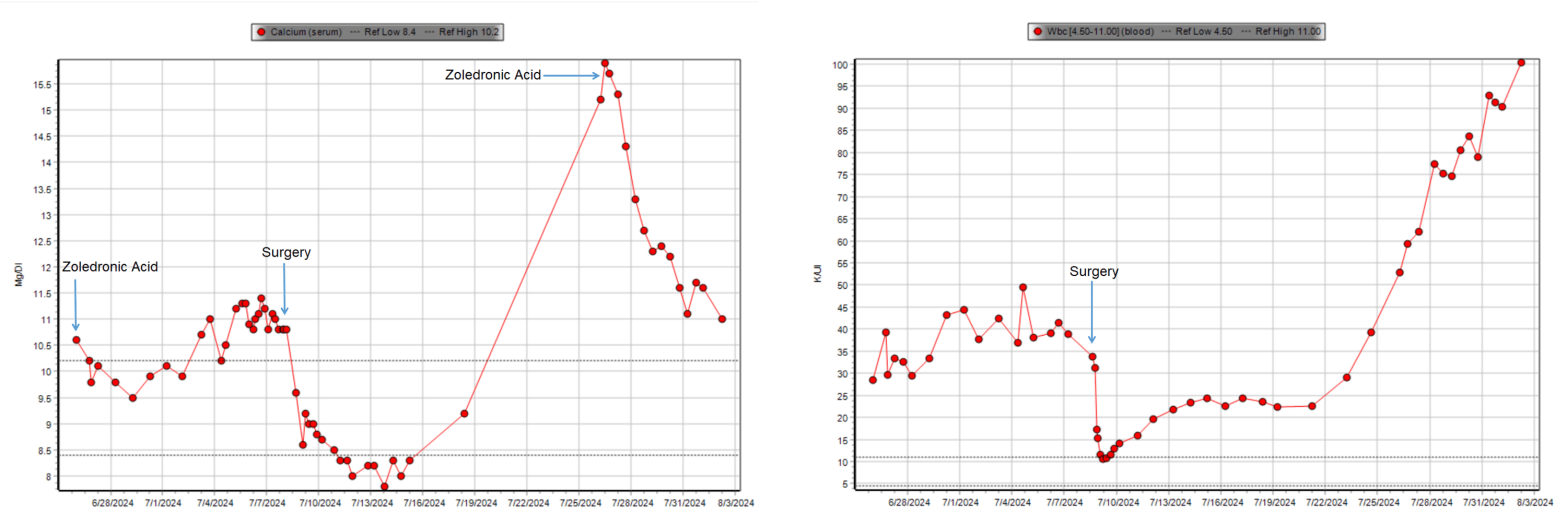
Laboratory trends for calcium and WBC Left: laboratory trend for calcium with arrows denoting key events including when the patient received zoledronic acid and surgery during his hospitalization. Right: laboratory trend for WBC with the arrow indicating when the patient underwent surgery during his hospitalization WBC: white blood cells

On admission, the patient's WBC value was 28.53 K/uL (4.50-11.00 K/uL) with a neutrophil count of 25120/uL (1100-7700/uL), which corresponded to 88.1% (41.0-85.0%) neutrophils. At its nadir, his WBC was 10.77 K/uL with a neutrophil count of 9100/uL, corresponding to 83.1% neutrophils postoperatively. Figure 2 displays the lab trends. The patient had rapid progression of his malignancy with evidence of metastatic disease and carcinomatosis as noted by interval PET/CT three weeks after his surgery showing large hypermetabolic masses in the pelvis, mesentery, peritoneum, and pelvic floor. He had developed an interval increase in size and volume of his primary mass with invasion into the anterior abdominal wall, multiple hepatic metastases, along with metastatic lymphadenopathy in the abdomen and pelvis.

Because of his aggressive disease and significant decline in functional status, the consensus at the multidisciplinary tumor board was that he was not a candidate for any further adjuvant or palliative chemotherapy or radiotherapy, and it agreed with the patient's and family's wishes to pursue comfort care. A month after his first dose, he required another dose of IV zoledronic acid 4 mg for rebound elevation in serum calcium. The following week, he was transitioned to hospice care and passed away shortly after. 

## Discussion

Hypercalcemia is a well-recognized paraneoplastic syndrome, frequently associated with malignancies such as breast, lung, or kidney cancers. Among the various mechanisms driving hypercalcemia in malignancy, PTHrP secretion is the most common, accounting for approximately 80% of cases [1,2]. Paraneoplastic hypercalcemia can arise via four primary mechanisms. First, humoral hypercalcemia of malignancy (HHM) caused by PTHrP secretion, leading to increased bone resorption and renal calcium reabsorption [18]. Second, osteolytic hypercalcemia due to direct bone destruction by metastases [2]. Third, vitamin D-mediated hypercalcemia caused by increased 1,25-dihydroxyvitamin D production by tumor cells [2]. And lastly, from ectopic PTH production [2].

In localized SCC of the bladder, cases of hypercalcemia are rare but have been reported and humoral hypercalcemia due to PTHrP appears to be the predominant mechanism [13-17]. PTHrP mimics the action of parathyroid hormone (PTH) by binding to the same type 1 PTH/PTHrP receptor, leading to increased osteoclastic activity and calcium release from bones, enhanced calcium reabsorption in the kidneys, and reduced phosphate reabsorption, contributing to hypercalcemia and hypophosphatemia [18]. Unlike PTH, PTHrP does not stimulate 1,25-dihydroxyvitamin D production, resulting in low or normal vitamin D levels despite hypercalcemia [2,18].

Patients with hypercalcemia due to PTHrP often present with nonspecific symptoms that can easily be mistaken for other conditions. In advanced cases, a hypercalcemic crisis can lead to profound dehydration, arrhythmias, or coma [2]. In the context of SCC of the bladder, these symptoms may overlap with those caused by the primary tumor, such as hematuria, pelvic pain, or obstructive uropathy [19]. 

Hypercalcemia in a patient with known or suspected malignancy warrants a thorough evaluation to confirm the etiology and guide management. Key diagnostic steps include obtaining confirmation of hypercalcemia, after adjusting for albumin. Ionized calcium measurement provides additional accuracy. Suppressed PTH (<20 pg/mL) is typical in malignancy-related hypercalcemia, differentiating it from primary hyperparathyroidism [1,2]. Elevated PTHrP levels confirm humoral hypercalcemia, although the diagnosis is usually apparent based on the clinical situation [1]. Vitamin D levels are typically normal or low in PTHrP-mediated hypercalcemia [2]. Contrast-enhanced CT or MRI can identify the primary tumor and assess for metastases. Whereas cystoscopy with biopsy remains the gold standard for diagnosing bladder cancer and confirming SCC histology [19]. 

Managing hypercalcemia in SCC of the bladder requires both correction of the immediate metabolic disturbance and treatment of the underlying malignancy. The primary goal is to rapidly lower serum calcium levels and address symptoms. Key interventions include IV hydration and bisphosphonates (e.g., zoledronic acid) which inhibit osteoclastic bone resorption [1]. Denosumab, a human monoclonal antibody to receptor activator of nuclear factor-κB ligand (RANKL), can be used as an alternative to bisphosphonates in the setting of renal dysfunction or hypercalcemia refractory to bisphosphonates [2]. Denosumab competitively inhibits RANKL binding to its receptor RANK, and thus inhibits osteoclasts and suppresses bone turnover [2]. Calcitonin provides rapid but short-lived reduction in calcium levels by inhibiting bone resorption and increasing renal calcium excretion [2]. Glucocorticoids are useful in cases of vitamin D-mediated hypercalcemia but less effective in PTHrP-driven cases [2]. 

Definitive treatment depends on controlling the underlying malignancy, which often requires a multimodal approach. Radical cystectomy is the mainstay of treatment for SCC of the bladder, though other options including neoadjuvant or adjuvant radiation therapy and other systemic therapies are available though not well established in their success [19]. In cases where the tumor is refractory to treatment, hypercalcemia often recurs, underscoring the importance of addressing both the metabolic and oncologic aspects of care. This was seen in our patient, as the normalization of calcium was not seen until initial de-bulking surgery with recurrence of hypercalcemia in the setting of incomplete resection and eventually metastatic disease. Because of such a poor prognosis, temporizing measures like bisphosphonates were appropriate for palliative measures. In general, the prognosis of SCC of the bladder is poor, as it is often diagnosed during the advanced stages of the disease [19]. Hypercalcemia itself further complicates the clinical course, contributing to significant morbidity and mortality even if adequately managed [20]. Early recognition and aggressive management of hypercalcemia can improve quality of life.

Interestingly, hypercalcemia in localized bladder cancer may also be associated with paraneoplastic leukocytosis. This phenomenon is related to tumor production of G-CSF which stimulates the development of mature neutrophils from hematopoietic progenitor cells [5]. It is diagnosed with marked leukocytosis with predominantly mature neutrophils, elevated serum G-CSF, positive immunohistochemical staining of tumor cells with anti-G-CSF antibody, and normalization of leukocytosis and serum levels of G-CSF after tumor removal [5]. The literature review revealed several cases demonstrating the overlap between hypercalcemia and leukocytosis paraneoplastic syndromes in bladder cancer that occur in both SCC and other bladder cancer histologies like TCC [10-17]. These cases also confer poor prognosis with rapid progression to death.

In this case, the original leukocytosis may have been from infectious etiology within the bladder mass because his WBC decreased only after intra-abdominal intervention, presumably from source control of the infection. His leukocytosis began to worsen at an increasing rate post-operatively despite being on antimicrobials after his partial cystectomy. Therefore, under the guidance of our infectious disease consult team, an extensive infectious workup including repeat imaging to look for new infectious sources was pursued, which was unremarkable. This concluded that his leukocytosis was likely due to the malignancy. Serum G-CSF was not measured in this case because it is not readily available at our institution and it would not have changed management.

## Conclusions

Paraneoplastic syndromes are uncommon in bladder cancer. Hypercalcemia due to PTHrP is a rare but severe complication of SCC of the bladder. Its diagnosis requires a high index of suspicion, especially in patients with nonspecific systemic symptoms alongside advanced bladder cancer. Effective management hinges on rapid correction of calcium levels and, ultimately, definitive treatment of the underlying malignancy. Paraneoplastic leukocytosis in bladder carcinoma is rare and can be associated with paraneoplastic hypercalcemia, and appears to be a particularly aggressive form of the disease. Given the rarity of this presentation, especially in bladder cancer, the constellation of signs of hypercalcemia, leukocytosis, and bladder mass should raise suspicion of a paraneoplastic syndrome associated with bladder cancer. Based on the current review of the literature, this is rare and unfortunately has a bad prognosis; further research and case reports are needed to enhance our understanding and improve patient outcomes. 

At our institution, endocrinologists are often called for the management and workup of hypercalcemia of malignancy and the objective of this case report is to highlight a unique presentation of hypercalcemia of malignancy and its possible association with another paraneoplastic syndrome of leukocytosis in the setting of bladder cancer. In cases where there is curative intent in the treatment of the underlying bladder malignancy, persistence and or recurrence of hypercalcemia or leukocytosis can be markers of disease progression. Treatment of hypercalcemia therefore plays an important palliative role, and leukocytosis in these cases may not represent infection but rather continued G-CSF production from the underlying malignancy.
